# Operationalizing Client-Centered Care: A Strategic Framework and Measurement Approach to Guide Sexual and Reproductive Health Programming

**DOI:** 10.9745/GHSP-D-24-00025

**Published:** 2025-12-31

**Authors:** Kathryn Church, Georgina Page, Sarindi Aryasinghe, Raman Shrestha, Inonge Wina Chinyama, Mary Morris, Kate Austen, Angela Argenziano

**Affiliations:** a Independent consultant, London, UK.; b MSI Reproductive Choices, London, UK.; c Imperial College London School of Public Health, London, UK.; d Children’s Investment Fund Foundation, Delhi, India.; e MSI Zambia, Lusaka, Zambia.; f NHS/Barts Health Trust, London, UK.

## Abstract

**Background:**

Promoting client-centered care (CCC) has been a long-standing goal for sexual and reproductive health (SRH) programs. MSI Reproductive Choices (MSI), a global SRH service delivery organization, set out to fully operationalize CCC delivery utilizing a new strategic framework and measurement approach.

**Development of the Approach:**

The framework was developed by a global technical group at MSI, based on a literature review, country pilots in Nepal and Zambia, and practical experience in service delivery and quality improvement. It is based on a socioecological model, recognizing that SRH client experience is determined by the behavior of the provider, the managerial support given to providers, and the broader organizational culture. The accompanying composite CCC metric assesses performance across these 3 levels annually using program monitoring tools. A digital results dashboard links to a suite of guidance and tools to support CCC improvement.

**Lessons Learned:**

SRH programs in 28 countries have been monitoring their progress on CCC since 2021, and a majority have made improvements in their CCC performance since then. Using the annual CCC results, global support staff and country managers have selected interventions based on local needs, including CCC training, client feedback systems, and client experience checklists. Implementation of a global staff engagement survey has been instrumental in CCC measurement, complementing preexisting client exit interview and quality audit processes. A global CCC monitoring process allows sharing of successes and positive practice, and staff have supported and championed CCC.

**Conclusion:**

Recognizing the critical influences of provider support and engagement as well as broader organizational culture has been pivotal in scaled operationalization of CCC within SRH programs. The simple framework and accompanying metric can be adapted to fit available tools and data systems in other institutions to support attainment of the highest standards of quality care and respect for client rights.

## Abstract (precis)

A strategic framework, measurement process, and suite of programmatic tools, piloted in Nepal and Zambia clinics and outreach services, helped to support operationalization of client-centered care in reproductive health and led to significant improvements in the client experience.

## Key Messages

- Delivering client-centered care (CCC) within reproductive health programs necessitates a multilevel framework to ensure organizational responsiveness, support for provider well-being and engagement, and ultimately the delivery of positive and empowering client experiences.
- In order to institutionalize a culture of care, measurement of CCC must move beyond client-provider interaction indicators to record progress at all levels of the system. Regular monitoring of CCC, supported by data dashboards and links to available tools and resources, can help programs improve CCC performance and the quality of reproductive health care.
- This article describes the CCC approach and tools developed and implemented by an international NGO working in multiple countries, which can be adapted by other reproductive health programs. The framework uses a socioecological model while the tools and resources leveraged are those evidenced as effective in supporting CCC and commonly available in the sector, such as client and provider surveys, observation checklists, training for frontline teams, client feedback systems, and client experience checklists.

## BACKGROUND

The promotion of client-centered care (CCC) has long been recognized as a key objective in the delivery of quality reproductive health services.^1^^,^^2^ Client-centered approaches, a form of patient-centered care, enable the delivery of human rights-based care, ensuring that all potential clients are treated with dignity and respect, that they make fully informed choices about their reproductive health, and that they are equal partners in decisions on their health care alongside health professionals. CCC in sexual and reproductive health (SRH) is both intrinsically important (all clients have the right to be treated with dignity and respect) and instrumentally important (through association with reproductive health care utilization and outcomes).^3^ Notably, positive SRH service experiences can lead to the successful use of contraceptives, in particular by decreasing the risk of premature method discontinuation, which leaves women at risk of unintended pregnancy.^4^^–^^10^ Provision of CCC is also associated with improved staff satisfaction, motivation and well-being, which are all contributing factors to sustaining a committed health workforce.^11^^,^^12^ Poor quality provision, including mistreatment by staff and concerns about privacy, can push clients to discontinue contraception or use unsafe care, which is a particular problem in the provision of abortion.^13^

In 2019, the international nongovernmental organization (NGO), MSI Reproductive Choices (MSI), set out to develop a CCC strategic framework and measurement approach with the goal of institutionalizing a culture of care within its SRH programs in low- and middle-income countries. The aim was to move beyond counseling and quality improvement interventions aimed solely at frontline providers, which generally have had mixed effects on sustained provider behavior change, and instead seek to address broader challenges including poor awareness among clients of what good client care should look like; insufficient client feedback or failure to act on it; didactic provider and supervisor training that was not sufficiently interactive; and evaluation metrics that did not support adequate accountability for all aspects of care quality, including CCC.

**FIGURE 1:**
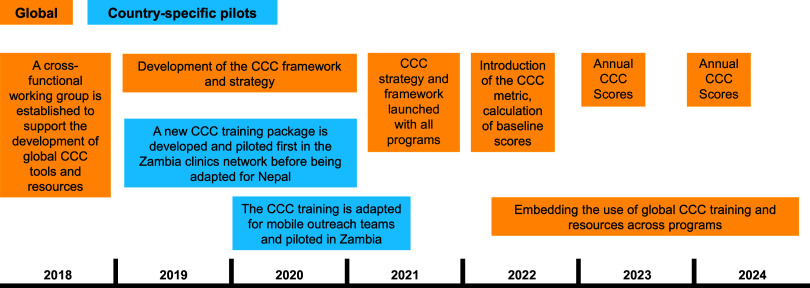
Timeline for Development and Implementation of MSI’s Client-Centered Care Framework and Metrics Abbreviation: CCC, client-centered care.

In this article we describe the process of developing the CCC strategic framework and a linked CCC measurement approach; a suite of tools that can support CCC strengthening; and the lessons learned on CCC operationalization. Case studies of pilot CCC training packages from Nepal and Zambia are also presented as part of the framework development, as their experiences and insights fed into the global approach (Figure 1).

The insights and learnings presented in this article come from different data sources. Client exit interview data used in the CCC metric received ethical approval from MSI’s independent Ethics Review Committee (MSI/ERC, 022-19A and 022-20AA). Nepal and Zambia case study data come from annual client exit interviews (ethical approval Zambia ERES, 2018-Oct-25; Nepal NHRC, 2052020P). Staff interview data from Nepal are from an unpublished process evaluation of CCC (NHRC, 2052020P; MSI/ERC 037-19).

## DEVELOPMENT OF THE STRATEGIC FRAMEWORK

To develop the strategic framework and measurement approach, a rapid scoping review was undertaken using PubMed (MEDLINE and PubMed Central) and hand-searching of selected SRH organizational websites. The aim was to identify effective CCC approaches and interventions, as well as existing strategy or measurement frameworks used by other organizations. Interventions identified predominantly focused on provider communication and behavior, and these had inconsistent results in their impact on client outcomes.^14^^,^^15^ Interventions with supervisors and managers were limited.^16^ Recent efforts to improve CCC measurement were helpful, but a focus on care processes at the client-provider interface meant they lacked an adequate framework to guide organizational or programmatic strategy.^17^^–^^22^

The literature scoping and MSI’s own practical experience as an SRH care provider indicated that systemic factors were key to sustained provision of CCC. This requires attention not only to common structural challenges in the health sector (such as client load and staff turnover) but also to the organizational culture underpinning the system.^23^^–^^27^ Tackling the ‘software’ of the health system and its management—factors like motivations, values, agency, commitment, and trust—is also instrumental in addressing complex behavioral and organizational change.^28^^,^^29^ Poor provider motivation is often driven by poor leadership and management.^30^^,^^31^ Studies also highlighted the need to minimize the burden on staff time during quality assessments; regularly evaluate quality (including clinical competence and interpersonal relations); use standardized tools; and ensure actions can be integrated into daily activities.^16^^,^^32^

Using these insights, a global CCC strategy team (involving 8 specialists on client care, provider support and motivation, human resource management, clinical assessment, program management, evaluation, and digital feedback systems) worked together to conceptualize a strategic framework to guide CCC. The structure and content of the framework needed to be simple in order to make the strategy understandable and the implementation operationalizable. We also sought to ensure that the approach built on and used preexisting quality improvement tools. Concurrent country pilots of a CCC training package in Nepal and Zambia also fed into the global strategic framework and measurement approach (Box 1). The framework and measurement approach underwent several rounds of review by operational and medical programming teams throughout the organization.

BOX 1 Client-Centered Care Training Pilots in Nepal and Zambia

Between 2019 and 2021, MSI undertook 2 CCC pilots, one in Nepal and the other in Zambia. Learnings and insights fed into the development of MSI’s global framework, with managers from both countries participating in the global working group. Pilot data is derived from client exit interviews from both countries, and in Nepal from interviews with 30 staff.

MSI Zambia (MSZ) aimed to strengthen CCC in both its clinic network (2 facilities) and its 10 outreach teams between 2019 and 2021. Interventions included improving key client touchpoints, with a focus on waiting times, privacy, and counseling quality; improving response to client feedback, including regular feedback review and use of ‘You said, We did’ boards; staff mentorship from both supervisors and peers; achievement recognition through ‘CCC stars’ awards; use of client journey role plays during staff meetings; and development of a once-per-month CCC training program for outreach teams.

Sunaulo Parivar Nepal (SPN), the Nepali NGO that implements MSI’s program in Nepal, strengthened CCC in 6 clinics in 2020 using an 8-module training course (online due to COVID-19); a walk-through client experience checklist; and client feedback guidance, tools, and resources to support action plans. SPN standard procedures were then adjusted, including ensuring greater privacy for young and unmarried clients; reduced fees (through fee waivers); and flexible opening hours for working clients. Practical actions were also taken to improve the management of wait times and more attention was given to ensuring a continuum of care through post-service follow-up.

**FIGURE 2:**
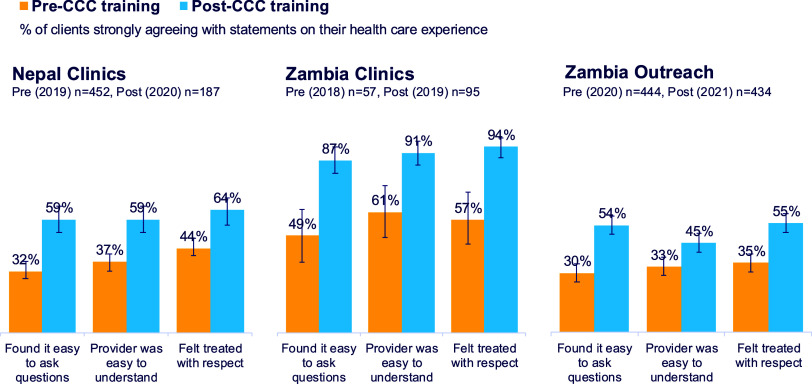
Select Client-Centered Care Indicators Before and After Training, Nepal and Zambia MSI Clinics and Outreach Services, 2018–2021 Source of data: Annual client exit interviews. Abbreviation: CCC, client-centered care.

In the 3 programs (MSZ clinics, MSZ outreach, and SPN clinics), program monitoring pre- and post-CCC training showed changes in 3 key measures of CCC: client found it easy to ask questions, client thought the provider was easy to understand, and client felt treated with respect (Figure 2). Most indicators showed significant improvement over the year of CCC focus. For example, the percentage of clients strongly agreeing that they felt treated with respect increased in Nepal clinics from 44% before the CCC intervention to 64% after the intervention, and in Zambia clinics from 57% to 94%. CCC attainment in MSZ outreach was lower overall than in the centers due to the very differing operating environment of outreach teams but still showed post-intervention improvements.

Program insights gleaned from the pilots included the importance of designing interventions that can be implemented on top of heavy workloads. Examples include:

- Once-per-month training slot for outreach teams
- Space for staff reflection on client feedback and team performance to allow time for necessary improvements
- Action-planning using client feedback
- Regular monitoring of CCC performance by team managers

Interviews with staff in Nepal indicated concerns about the time investments needed to deliver CCC, although time was freed up by limiting head-office managerial demands to a daily request, thus reducing service disruptions. Management staff also emphasized the importance of involving the whole organization in CCC, not only providers:

*… Overall the impact of client-centered care is really big … it looks very small, and one may think it is just another responsibility, but if we follow it, and provide better services it can have a great impact … in a sector like ours, CCC involves the guard to the manager, from line manager to director or also the country director … This is the responsibility of all. This is not only one single person’s responsibility.*

**FIGURE 3:**
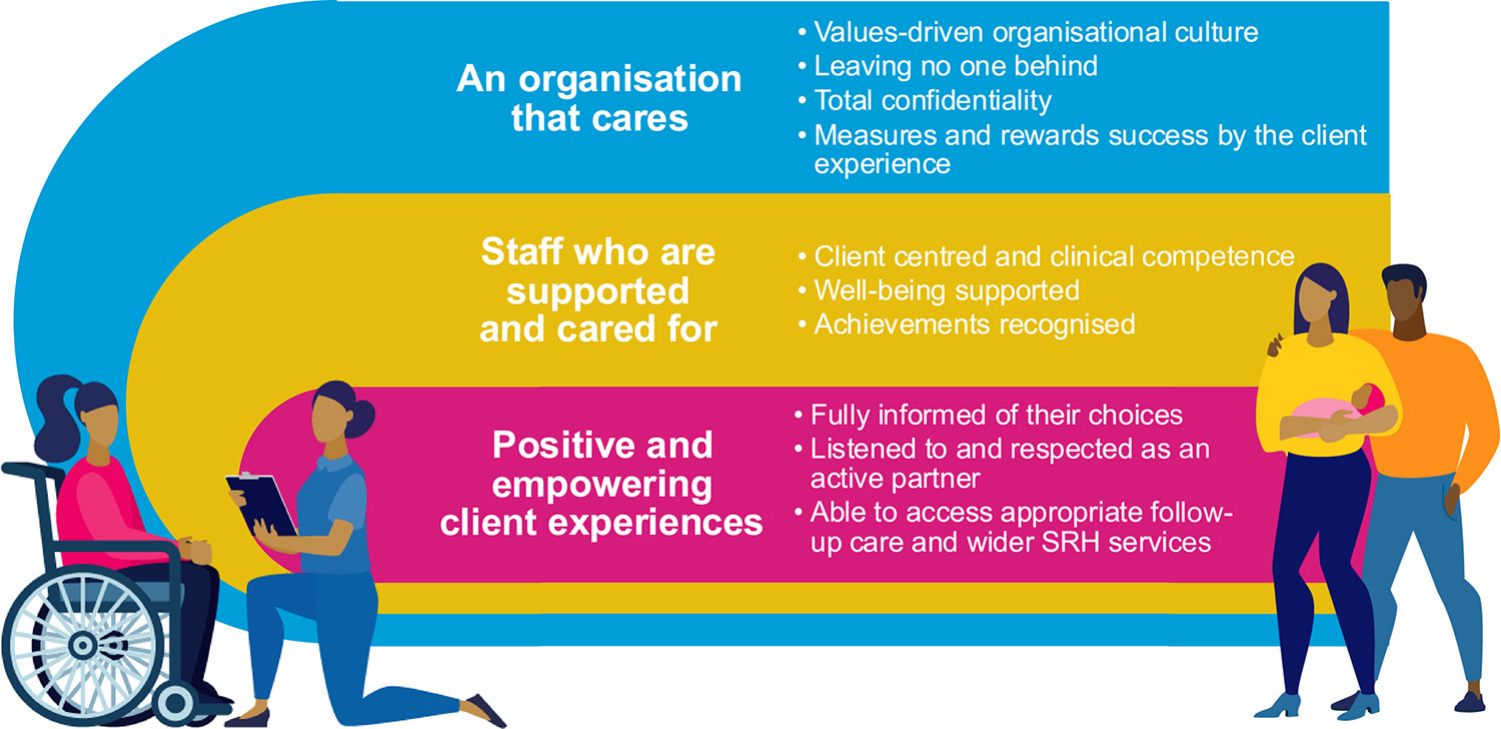
MSI Strategic Framework to Guide Client-Centered Care Abbreviation: SRH, sexual and reproductive health.

Following the experiences and insights gained from the Nepal and Zambia pilots, the global team opted to develop a 3-layered model that emphasized the role of the whole organization in delivering CCC. The new CCC framework is shown in Figure 3. Its structure is derived from socioecological models that recognize how outcomes and behaviors are driven by broader sociocultural, political, or economic influences. It values attributes of care that are essential to the SRH sector, including positive and empowering client experiences to support women in their rights to reproductive autonomy, as well as an organizational culture that respects total confidentiality and strives for equitable access to care, ‘leaving no one behind.’ To support cultural change within a results-driven organization, it also measures success not only by numbers of clients served but also by the client experience. Perhaps most importantly, it draws attention to the critical role of health providers, guiding programs to ensure that they are supported and cared for, as well as training in CCC competencies. Provider support is particularly critical in environments where SRH is highly stigmatized or where health workers face backlash from family and community members.^33^

### Measurement Approach

**FIGURE 4:**
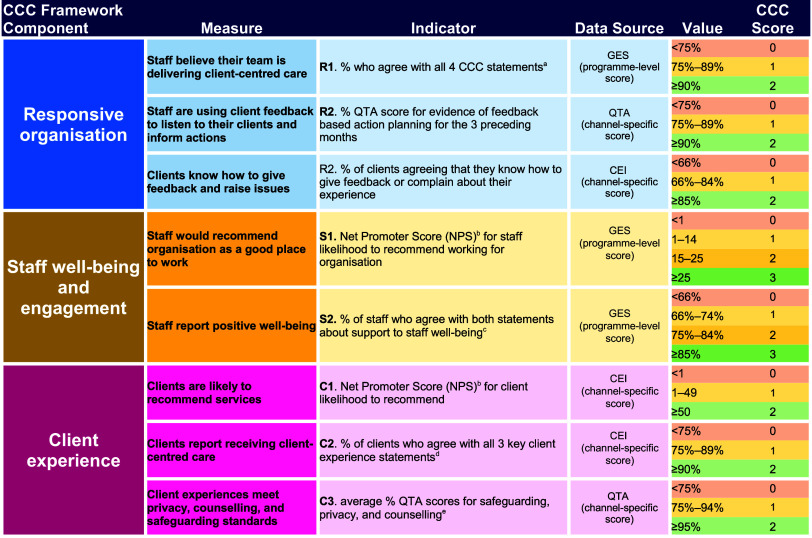
MSI Client-Centered Care Indicators and Scoring Mechanism Abbreviations: CCC, client-centered care; CEI; client exit interview; GES, global staff engagement survey; QTA, quality technical audit. ^a^ The 4 CCC staff statements are: (1) I believe my team delivers a high-quality service to its clients; (2) I feel confident clients receive the support they need to choose services they feel is right for their health and well-being; (3) My team regularly discusses ways to improve the service for our clients; and (4) My colleagues show concern for the well-being of the clients we support in a caring and meaningful way. ^b^ Net Promoter Score is calculated from the proportion of respondents rating their likelihood to recommend at 9 or 10 out of 10, subtracting the proportion rating their likelihood at 6 or less out of 10. Measured as an integer from −100 to +100. ^c^ The 2 staff well-being statements are: (1) My employer cares about its staff; and (2) My employer gives me the help I need to manage my well-being effectively. ^d^ The 3 CCC client experience statements are: (1) I was treated with respect during my visit; (2) I understood what the provider was telling me; and (3) I found it easy to ask the provider questions. ^e^ Safeguarding score (1 item); privacy score (2-3 items); counseling score (about 15 items). Numbers of items for each score varies by service channel. Note: For all scaled items, agreement scores include both ‘agree’ and ‘strongly agree’ categories.

A strong measurement approach is critical to track progress in CCC efforts, to manage performance and reward success. To avoid a large reporting burden, we opted to select indicators of CCC performance rather than aiming for a comprehensive assessment of each level of the framework. Figure 4 details all the indicators and resultant CCC scoring bands. The CCC metric complements other core quality metrics used in assessment of country program performance.^34^^,^^35^

Existing monitoring and evaluation mechanisms were reviewed and updated to ensure the collection of the necessary CCC data. Indicators were drawn from 3 preexisting quality monitoring tools: a client exit interview survey, conducted annually among a representative sample of clients and service delivery points; a quality technical audit, conducted annually in a representative sample of service delivery points; and a digital staff global engagement survey, sent to all staff. The staff engagement survey was newly introduced in 2021 and was instrumental in comprehensive measurement of CCC. While a research company was hired to run the staff survey, similar tools are publicly available.^36^

The metric therefore relies on self-reported data from clients and providers as well as observed data from external clinical auditors. Each of these data collection processes has their own quality assurance processes to ensure the validity and reliability of the data collected.

A new global ‘Client Experience’ team helped program managers to operationalize the concepts, set goals, and track progress. The strategy was communicated to senior leadership teams across 28 countries through multiple channels including webinars, in-person presentations, and regular technical support meetings.

**FIGURE 5:**
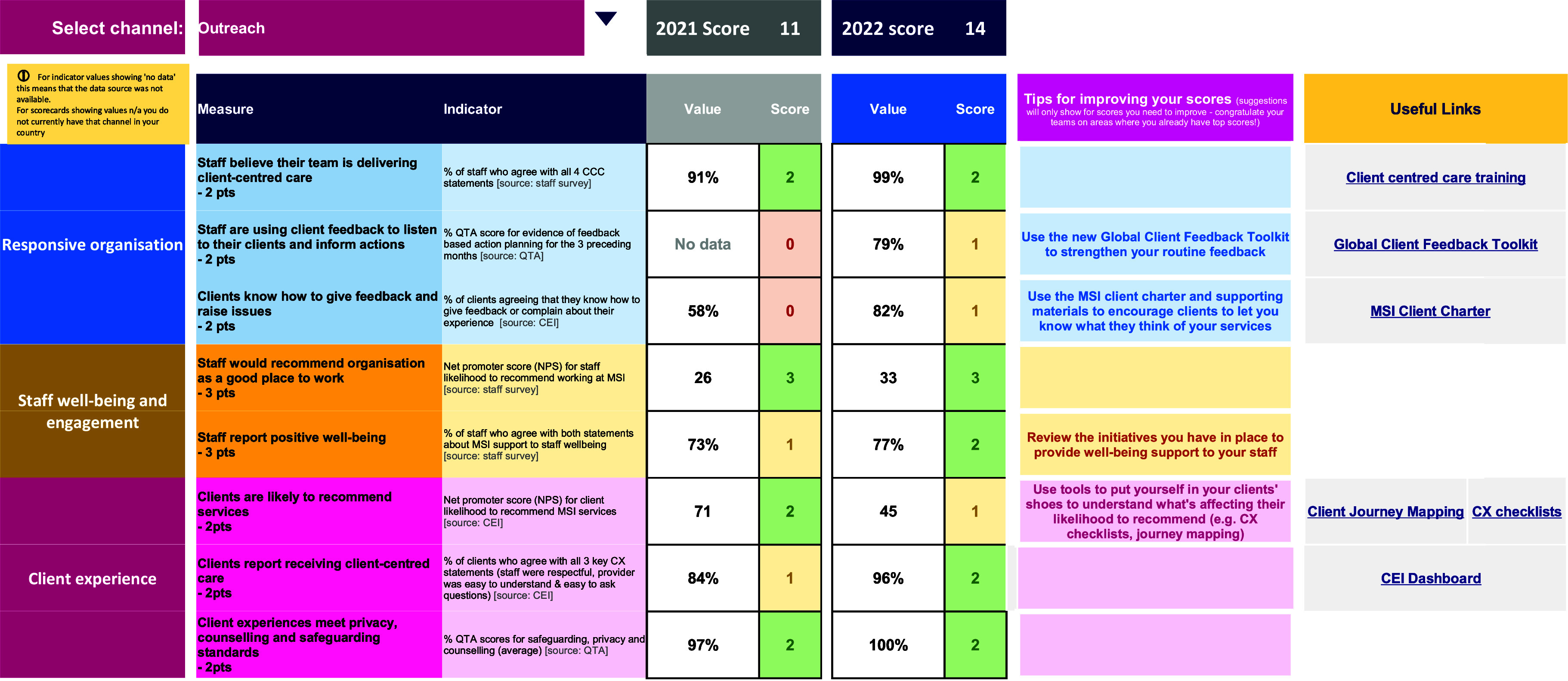
Example of MSI’s Client-Centered Care Dashboard (anonymous country) Abbreviations: CCC, client-centered care; CEI, client exit interview; CX, client experience; QTA, quality technical audit.

Each country program receives their CCC scores annually, disaggregated by service delivery channel; total country scores are weighted by client volume in each channel. Teams review their CCC score via a global dashboard (Figure 5).

Low and medium value scores trigger suggested actions (‘tips for improving your scores’) as well as links to relevant tools that can be used to improve performance over the following year (Box 2).

BOX 2 Tools and Guidance to Support Strengthening of Client-Centered Care

The following guidance and tools can be used and adapted to support CCC strengthening. Most are publicly accessible. Items with an asterisk (*) are available upon request from the corresponding authors.

**Organization-wide policies and strategies:**

- Code of conduct (signed by all staff, supported by online and in-person training*)
- Safeguarding guidance, including adult safeguarding policy
- Data privacy guidance* (MSI’s guidance aligns with UK General Data Protection Regulation [GDPR] standards) to ensure that client and staff data are kept fully confidential

**Clinical and nonclinical training:**

- Comprehensive clinical guidance, competency assessments, and audit process*
- Values Clarification and Attitude Transformation (VCAT) workshops to support positive attitudes to clients accessing SRH care and to reduce stigma
- CCC training modules* for frontline teams exploring key CCC themes and how these can be practically addressed during the delivery of health care services
- ‘Providers Share’ workshops for abortion providers to address their own stigmatizations

**Client-facing materials and support:**

- Client Charter – MSI’s full resource pack* includes editable posters and video, describes values of client-centered health care, and encourages client to hold the organization accountable and provide feedback
- ‘Choice Counsellor’ (digital contraception decision aid), which provides clients with information on their method options and where to seek further advice and support
- ‘Choice Kit’ (contraceptive method demonstration kit) and counseling flipcharts, which support providers to deliver client-centered contraceptive counseling

**Resources for monitoring the client experience:**

Feedback review and action-planning form part of the organizational culture score in the CCC framework and are emphasized in CCC training. Tools include:

- Client exit interviews provide a detailed annual overview of the profile of clients and their health care experiences
- Global client feedback toolkit supports service teams to implement simple mechanisms for collecting routine feedback from clients and helps them use and respond to this data
- Client experience checklists* empower staff and managers to put themselves in the shoes of clients and understand their full health care journey, including potential pain points, to identify areas for improvement
- Mystery client survey protocol* provides objective assessment of client experiences and client-provider interactions

In 2021, the year in which the CCC framework and metric were first introduced to countries, scores ranged from 5 to 17 out of a possible 18 points. In 2022, the second year of the strategy rollout, almost half (13/28) of countries had been able to achieve improvements in their overall program score (there was no change in 5 countries and a decrease in scores in 10 countries, with the latter driven by low staff engagement data). Improvements continued into 2023, with 19 of 28 country programs increasing their scores year-on-year (6 countries saw no change and only 3 had decreases). Common areas of improvement included the strengthening of the collection and use of client feedback and some small but positive shifts in staff engagement and well-being.

## LESSONS LEARNED

### Key Contributors to Scaled Operationalization

A simple multilevel framework, recognizing the role of organizational culture in provider behavior and client interactions, has enabled effective communication to managers at all levels of the organization. Linking the framework to performance measurement and then to available tools has been particularly important.

A standardized CCC performance metric utilizing existing data sources and a performance dashboard has been essential. The annual review of CCC data by in-country teams alongside other performance metrics, such as the numbers of people reached with services, helps ensure equal value is placed on CCC. Annual data collection activities facilitate measurement, namely client exit interviews, clinical audits, and a new digital global staff survey. The data dashboard allows programs to review CCC performance over time, benchmark against other similar countries, understand their areas of weakness, and link to accessible tools and resources to improve performance. It also encourages productive sharing of successes and positive practice.

A data-driven approach puts the onus on middle and senior management to own and champion CCC, prioritize its discussion in periodic reviews, and include CCC in strategic planning and budgeting. Country program teams are supported by a global CCC team to understand their dashboard scores, and it has been important to allow time to embed processes and build capacity to do this.

A focus on improving client-friendly feedback mechanisms has allowed effective action-planning at facility/team level. Regular client feedback enables frontline teams to understand client complaints and plan actions on a regular basis. Other contributing factors have included having assigned staff for CCC at global and country levels and the development of provider training on ‘soft skills’ (non-clinical aspects of service provision), including communication skills, empathy, and client rights.

### Staff Perspectives on CCC Implementation

A global staff engagement survey was initiated in 2022, completed by 4,205 staff members from 36 countries (response rate of 77%). Results indicated very strong consensus on prioritization of CCC. Nearly all staff agreed or strongly agreed that the organization delivers high-quality service to its clients (96%), felt confident that clients receive the support they need to choose services they feel is right for their health and well-being (96%), felt colleagues show concern for client well-being in a caring and meaningful way (94%), and that teams regularly discuss ways to improve services for clients and stakeholders (94%).

Work is ongoing to support country teams to review the staff engagement data and create action plans from their staff survey data, including to address staff well-being, provide mental health support, and training on diversity, equity, and inclusion (DEI). It is also important to note that staff engagement is also determined by broader good management practices, including policies on remuneration, recognition schemes, workload management, personal development and mentorship, and DEI policies.

### Ongoing Challenges

Challenges include the competing priorities of health service managers, including the need to ensure high and efficient volumes of service delivery with clinical excellence, which can then lead to a deprioritization of provider support and well-being within tight budgets. The value of provider support investments can also be hard to demonstrate when their impacts are not immediately evident (i.e., there may be no direct effects on service efficiency, health outcomes, or even client experience in the short term). Furthermore, delivering organizational culture change takes time and can be challenging to justify when health programs require ‘quick wins.’ The annual composite metric also has its limitations; score interpretations usually require teams to draw on further data, experience, or enhanced monitoring to interpret changes in their scores and inform actions.

### Expanding CCC Support to the Public Sector

MSI’s CCC framework and measurement approach can be adapted by other organizations and public-sector authorities. The framework and tools are not designed to be prescriptive or all-encompassing, and the CCC metric does not attempt to comprehensively measure CCC. Instead, adaptation should focus on the process of CCC strengthening. This can include, for example, identifying CCC champions; measuring some aspects of CCC regularly at facility level; and ensuring that the whole organization measures success by client experience in addition to the number of clients served or the number of services delivered.

The resources required to deliver CCC may depend on how health organizations currently monitor quality. Clinical audits are commonplace within public health systems and could be adapted to ensure observation of client-centered behaviors or organizational practices that uphold client rights. Annual representative client exit surveys are resource-intensive, but standardized tools and protocols exist and can be adapted.^37^^–^^39^ Improvements in technology may allow increased use of digital client feedback systems for quality improvement (and potentially remove the need for representative surveys). This could entail the use of red-flag incident reporting (e.g., incidents of disrespect, abuse, privacy violations, or contraceptive coercion). Digitalization has also enabled the rapid rollout of MSI’s annual staff survey, and these approaches can be easily adopted by other health organizations at low-cost.^36^

## FUTURE EVOLUTION OF THE APPROACH

Future work may involve iterations of the CCC metric to ensure all core attributes of care are covered. Given an increasing body of evidence on the potential for coercive practices and provider bias in contraceptive provision,^40^^,^^41^ greater attention to the measurement of coercion and/or reproductive autonomy may be needed. This can be informed by recent work on measures of fully informed choice and reproductive autonomy.^42^^,^^43^ Work is also needed to integrate equity measures (on poverty and disability) into the metric, while adaptation of the measure for different models of service delivery could be helpful (e.g., for community health workers). Improved measures of client confidentiality and privacy may also be helpful given their importance as a key aspect of CCC to both clients and providers. Many programs are also striving to increase client voice and participation in health service planning and improvements. This means using participatory approaches to involve clients in the co-creation of improvements to direct service delivery and exploring how health delivery organizations can support and encourage social accountability. Both approaches could be incorporated into a CCC framework.

While the Zambia and Nepal case studies in Box 1 show encouraging signs of CCC’s impacts on client outcomes, further evaluation work would also be helpful to demonstrate that the CCC strategic framework, measurement approach, and suite of tools make a meaningful difference to client experience and provider and institutional outcomes, both in the short and long term. A larger controlled study to evaluate the impact of strengthened CCC in a program with previously low performance on client experience and/or staff engagement indicators would be helpful for budgetary advocacy. Further research on the impacts on specific client groups, in particular adolescents, may also be helpful.

## CONCLUSIONS

The CCC framework, measurement approach, and tools presented in this program case study are successfully supporting NGO-run SRH programs in low- and middle-income countries in their efforts to improve the quality of care provided to their clients. The simplicity of the framework and metric, linked to a range of available tools and resources, has allowed managers to identify and act on opportunities to strengthen the delivery of CCC in their contexts according to their specific needs. The widespread acceptance of the framework and positive feedback from managers and providers from a diverse range of country settings has been encouraging. This initiative complements the work of other organizations attempting to promote person-centered approaches to health care, including in the SRH health sector.^44^^,^^45^ These efforts continue to be important as recent evidence documents encroachments on or violations of human rights within reproductive health programs across a range of country contexts, including low-, middle-, and high-income settings.^40^^,^^46^^–^^51^

A frequently asked question is whether CCC is feasible within busy health programs, where providers are rushed off their feet and face overwhelming demands from clients. Experience from MSI services, on the whole, shows that it is possible to be client-centered within a busy service environment. Being respectful and kind to clients does not necessarily require more time—rather, it necessitates a culture in which the provider feels equally cared for and respected by their employer. Engendering this culture of care does not necessarily mean having to pay providers more (though this may be helpful!), but it does involve good management practice in which providers feel rewarded and recognized for their work. This, in turn, needs to be supported by an institutional culture that places the client experience (and in the SRH sector, the fulfillment of human rights) at the core of their organization. Further effort is now needed to support all health programs (public, private, nonprofit, faith-based) to foster such supportive management practices in order to grow a culture of care in their organizations.

## Notes

**Peer Reviewed**
